# Interactions among oscillatory pathways in NF-kappa B signaling

**DOI:** 10.1186/1752-0509-5-23

**Published:** 2011-02-03

**Authors:** Yunjiao Wang, Pawel Paszek, Caroline A Horton, Douglas B Kell, Michael RH White, David S Broomhead, Mark R Muldoon

**Affiliations:** 1 Mathematical Biosciences Institute, The Ohio State University, Jennings Hall, Columbus, Ohio 43210, USA; 2 Centre for Cell Imaging, School of Biological Sciences, Bioscience Research Building, Crown Street, Liverpool, L69 7ZB, UK; 3 School of Chemistry and The Manchester Interdisciplinary Biocentre, University of Manchester, 131 Princess Street, Manchester M1 7DN, UK; 4 School of Mathematics, Alan Turing Building, University of Manchester, Manchester M13 9PL, UK

## Abstract

**Background:**

Sustained stimulation with tumour necrosis factor alpha (TNF-alpha) induces substantial oscillations—observed at both the single cell and population levels—in the nuclear factor kappa B (NF-kappa B) system. Although the mechanism has not yet been elucidated fully, a core system has been identified consisting of a negative feedback loop involving NF-kappa B (RelA:p50 hetero-dimer) and its inhibitor I-kappa B-alpha. Many authors have suggested that this core oscillator should couple to other oscillatory pathways.

**Results:**

First we analyse single-cell data from experiments in which the NF-kappa B system is forced by short trains of strong pulses of TNF-alpha. Power spectra of the ratio of nuclear-to-cytoplasmic concentration of NF-kappa B suggest that the cells' responses are entrained by the pulsing frequency. Using a recent model of the NF-kappa B system due to Caroline Horton, we carried out extensive numerical simulations to analyze the response frequencies induced by trains of pulses of TNF-alpha stimulation having a wide range of frequencies and amplitudes. These studies suggest that for sufficiently weak stimulation, various nonlinear resonances should be observable. To explore further the possibility of probing alternative feedback mechanisms, we also coupled the model to sinusoidal signals with a wide range of strengths and frequencies. Our results show that, at least in simulation, frequencies other than those of the forcing and the main NF-kappa B oscillator can be excited via sub- and superharmonic resonance, producing quasiperiodic and even chaotic dynamics.

**Conclusions:**

Our numerical results suggest that the entrainment phenomena observed in pulse-stimulated experiments is a consequence of the high intensity of the stimulation. Computational studies based on current models suggest that resonant interactions between periodic pulsatile forcing and the system's natural frequencies may become evident for sufficiently weak stimulation. Further simulations suggest that the nonlinearities of the NF-kappa B feedback oscillator mean that even sinusoidally modulated forcing can induce a rich variety of nonlinear interactions.

## Background

Nuclear factor kappa B (NF-*κ*B) transcription factors are critical to the control of response to cellular stress and are also involved in the regulation of cell-cycle/growth, survival, apoptosis, inflammation and immunity [1-5]. They are dimeric molecules, composed of either homo-or hetero-dimers, with the most common form being the RelA:p50 hetero-dimer.

In resting cells, NF-*κ*B dimers are sequestered in the cytoplasm by members of a family of molecules, the inhibitors of *κ*B or I*κ*Bs. When the cells are stimulated with tumour necrosis factor alpha (TNFα) certain kinases, the IKKs, are activated and phosphorylate I*κ*Bs, marking them for degradation. The degraded I*κ*Bs then release NF-*κ*B dimers which are free to translocate into the nucleus, where they bind to specific sequences in the promoter or enhancer regions of target genes, including those for I*κ*Bα and the zinc finger protein A20 [6,7]. Newly synthesized I*κ*Bα migrates to the nucleus, binds to NF-*κ*B dimers and removes them from the nucleus, while A20 protein stays in cytoplasm and represses the activity of TNFα receptors [8]. Hence the NF-*κ*B system includes at least two negative feedback loops, one involving cytoplasmic sequestration mediated by I*κ*Bα and another involving A20.

Coupled negative feedback loops are known to have the potential to support oscillations [9,10]. Biological evidence suggesting NF-*κ*B oscillations was reported by Hoffmann *et al*. [11], who did population-level studies of I*κ*Bα-/-embryonic fibroblasts using electro-mobility shift assays, and also observed by Nelson *et al*. [12] in the cellular concentration and nuclear: cytoplasmic localisation of I*κ*Bα and RelA-fluorescent fusion proteins in single SK-N-AS cells. Single cell time-lapse imaging data showed persistent cycling of NF-*κ*B localisation between the cytoplasm and nucleus of SK-N-AS cells in response to continual TNFα stimulation [12,13].

The expression of genes regulated by NF-*κ*B is tightly coordinated with the activities of many other signalling and transcription-factor pathways [14-18] including the p53 signalling pathway. Though the canonical NF-*κ*B signalling pathway has been studied extensively, the existence and mechanisms of the interactions between the NF-*κ*B pathway and other signalling pathways are unclear.

Ashall *et al*. [19] reported that different patterns of pulsatile stimulation of the NF-*κ*B system lead to different patterns of NF-*κ*B dependent gene expression, supporting the view that the frequency of the oscillations may have a functional role. It is therefore important to understand how the system's response frequencies are influenced by interactions with other oscillatory pathways. If both the NF-*κ*B network and its couplings to other other oscillatory pathways were purely linear, then it would be straightforward to use the machinery of transfer functions to characterise their interactions. In particular, one would expect the power spectrum of a periodically stimulated system to have its power concentrated at the forcing signal's frequency and its harmonics. But the NF-*κ*B network is a highly nonlinear system of coupled chemical reactions and hence, as we will demonstrate in modelling studies below, its response to periodic stimulation can include complex interactions between the intrinsic negative feedback oscillator and the stimulus. In what follows we work with a deterministic model first described in [19] and examine the power spectral densities of the time courses of NF-*κ*B localisation when the model is subjected to two types of periodic signals: trains of rectangular pulses and sinusoidal signals. Both sorts of stimulus are intended as a proxies for the influence of other oscillatory pathways on the core NF-*κ*B feedback loop and we find that such interactions can produce a rich variety of nonlinear dynamical behaviour. Our results on sinusoidal forcing are in broad agreement with those of Fonslet *et al*. [20], who applied a related family of sinusoidal stimulation protocols to a reduced model of the core NF-*κ*B oscillator originally developed in [21].

## Results and Discussion

### Experimental phenomena and time series analysis

**Figure 1:**
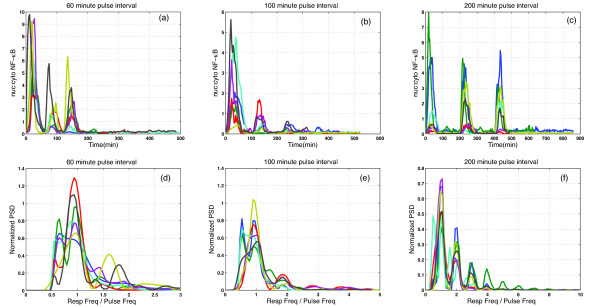
**Power spectra of single-cell data**. Panels (a), (b) and (c) show time courses of the ratio of nuclear-to-cytoplasmic fluorescence recorded from single cells while panels (d), (e) and (f) show the corresponding power spectral densities, normalized to have unit area beneath the curve. Note that the frequencies in panels (d)-(f) are measured in units of the stimulus frequency and so the horizontal scale varies from panel to panel.

We begin by analyzing the time courses of the localisation of fluorescently tagged NF-*κ*B in single cells subjected to TNFα stimulation (see [22] for details). The panels in the top row of Figure 1 show the ratio of nuclear-to-cytoplasmic fluorescence for three patterns of stimulation. In all cases the cells received three strong (10 ng/ml) pulses of TNFα, each of five minute duration. These pulses were separated by various inter-pulse intervals—55 minutes for panel (a),95 minutes for panel (b) and 195 minutes for panel (c)—resulting in net stimulus periods of 60, 100 and 200 minutes. The first of these is rather shorter than the observed period of the oscillations induced by constant TNFα stimulation, which is close to 100 minutes.

The bottom row of Figure 1 shows the corresponding power spectral densities and makes the point that the intrinsic oscillation—the one induced by constant stimulation—does not appear to have been excited by these pulsed stimulation protocols: in all cases the peaks in the power spectral density appear at multiples of the stimulus frequency. In mathematical terms, the cells respond as though they have been perturbed away from a stable resting state and do not offer evidence of any more complex internal dynamics. Nonetheless, these data provided crucial input to the development of the models in [19]. In particular, note that the amplitude of the response to the second and third pulses of stimulation varies as a function of the inter-pulse period, being somewhat reduced in panels (a) and (b), but not in (c): this constrains the rate at which the model should relax toward equilibrium.

One possibility is that the pulsing signal is too strong and suppresses the response at the natural frequency. In the following section we report in numerical experiments, based on the deterministic model in [19], through which we investigate this possibility by simulating the system's response to a periodic train of square pulses with strength varying from 0 to 10 ng/ml and inter-pulse intervals ranging from 20 to 295 minutes.

### Model Introduction and Bifurcation Analysis

**Figure 2:**
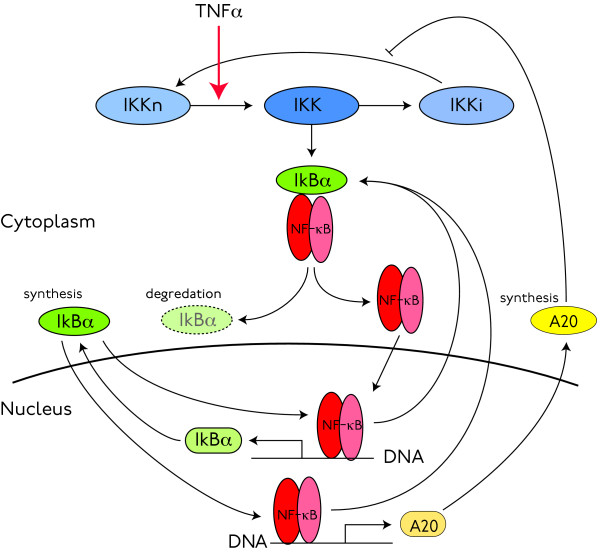
**The deterministic, two-loop model of **[19]. Proteins are shown as coloured ovals, while mRNA's are shown round-cornered rectangles. In addition to the direct negative feedback through which nuclear NF-*κ*B leads to the production of I*κ*B*α*, and thus to its own inactivation, the model also includes an indirect negative feedback mediated by A20's influence on the signal transduction machinery.

In this section, we introduce a deterministic mathematical model developed in [19]. It includes two coupled negative feedback loops—one for NF-*κ*B-I*κ*Bα interactions and another that models the dynamics of A20—as is illustrated in Figure 2. Here A20 acts by modifying the activity of I*κ*B kinase (IKK), which transduces the TNFα signal by phosphorylating I*κ*Bα, thus initiating the production of free NF-*κ*B. IKK is assumed to exist in one of three states: neutral IKK (denoted IKKn, a state ready for activation), active IKK (the state capable of phosphorylating I*κ*Bα) and inactive IKK (IKKi, a state unable to phosphorylate I*κ*Bα and also incapable of being activated by the TNFα signal). TNFα activates IKK by transforming neutral IKK into active IKK, which then acts to liberate NF-*κ*B. Active IKK is assumed to convert to its inactive form spontaneously, with linear kinetics. In the absence of A20, this inactive IKK would then transform back into neutral IKK, also with linear kinetics. A20 down-regulates NF-*κ*B activity indirectly, by retarding the transformation of inactive IKK to the neutral form: Equations (1) provide a simple model for these dynamics:

(1) $$\begin{array}{c} IK{K^{\prime }}_{n}=k_{p}\cdot IKK_{i}\cdot \left(\frac{k_{bA20}}{k_{bA20}+A20\cdot T_{R}}\right)\\ \;-T_{R}\cdot k_{a}\cdot IKK_{n}\\ IKK^{\prime }=T_{R}\cdot k_{a}\cdot IKK_{n}-k_{i}\cdot IKK\\ IK{K^{\prime }}_{i}=-k_{p}\cdot IKK_{i}\cdot \left(\frac{k_{b}{}_{A20}}{k_{bA20}+A20\cdot T_{R}}\right)\\ \;+k_{i}\cdot IKK. \end{array}$$

Here we have suppressed the time dependence of the concentrations of the various forms of IKK and of the A20 protein. The k_* _are fixed parameters and *T_R _*is a parameter indicating the presence or absence TNFα stimulation: *T_R _*= 1 when the system is being stimulated with 10 ng/ml TNFα and *T_R _*= 0 otherwise. This is a simplification: TNFα does not act directly on IKK, but rather produces its effect through a cascade of chemical reactions that begins when TNFα binds its receptor at the cell surface.

Following Ashall *et al.*, we treat only the final stages of this chain. Thus when we model strong stimulation by setting *T_R _*= 1, we abstract away a great deal of transduction machinery. In the studies that follow we will want to explore the consequences of weaker stimulation (TNFα concentrations of 10 ng/ml are orders of magnitude higher than physiological levels: Matalka *et al*. [23] report measurements from various tissues in healthy mice and found concentrations in the range 1-5 pg/ml while Prabha *et al*. [24] studied the plasma of healthy human subjects and found TNFα concentrations on the order of 100 pg/ml.) and it is natural to generalize the role of *T_R_*, allowing it to vary across the interval 0 ≤ *T_R _*≤ 1. In light of the modelling assumptions discussed above, one should not imagine that *T_R _*depends linearly on the concentration of TNFα, but only that *T_R _*increases monotonically with dose.

**Figure 3:**
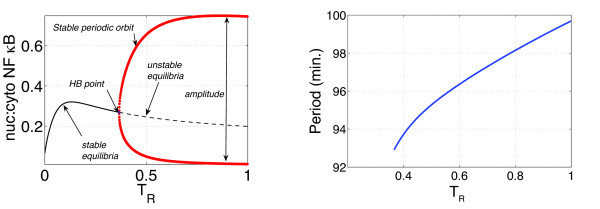
**Qualitative behaviour as a function of *T_R_***. At left, a bifurcation diagram showing how the oscillatory response depends on *T_R_*. The solid black curve in the region 0 ≤ *T_R _*≤ T_* _represents a branch of stable equilibria-steady, non-oscillating solutions-while the dashed black curve for *T_R _*>T_* _indicates a branch of unstable steady states. The red curves show the limits-the peak and trough values-of the stable oscillatory responses that exists for these values of *T_R_*. The panel at right shows period of the oscillation as a function of *T_R_*.

The model of Ashall *et al*. captures two important dynamical features observed in single cell data [12,19]: (i) continuous stimulation with high doses of TNFα leads to sustained oscillations with a period of around 100 minutes and (ii) pulsatile stimulation with the same high concentration of TNFα leads—as illustrated in Figure 1—to entrainment of the response by the pulsing signal. Given that continuous, strong stimulation induces sustained oscillations, one is prompted to ask how the existence of these oscillations depends on the strength of stimulation. We addressed this question by doing a bifurcation analysis using *T_R _*as the parameter: Figure 3 is the resulting diagram. The model has a Hopf bifurcation (HB) at *T_R _*= T_* _≈ 0.366, which means that for *T_R _*>T_* _the ratio of nuclear-to-cytoplasmic NF-*κ*B concentration exhibits sustained oscillations, but for *T_R _*<T_* _only damped oscillations. A forthcoming paper, Wang *et al*. [25], will present a comprehensive survey of the bifurcation structure of this model, but here we will concentrate on spectral analysis of the responses.

### Numerical experiments

All the numerical experiments reported here begin from the same initial condition, whose preparation is described in the Methods section. We simulated the consequences of two TNFα stimulation protocols: pulse-like stimulation similar to that used in Nelson's experiments and sinusoidally modulated stimulation.

#### Pulsed Stimulation

From the experiments and simulations in [19], we know that stimulation with a finite train of three strong pulses tends to entrain the cell's response. In this section, we first show that when subjected to a long periodic train of strong pulses, the model's response frequencies are also entrained, but that when the model is driven by sufficiently weak periodic pulse trains, various resonance phenomena connected to the underlying natural oscillations become observable.

**Figure 4:**
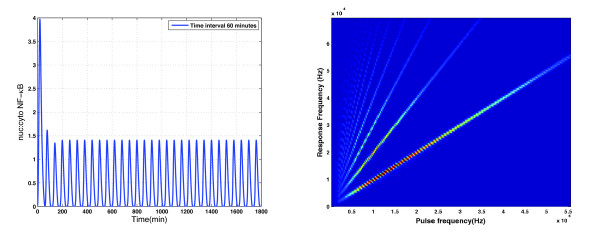
**Periodic stimulation with strong pulses**. The panel at left shows the model's response to sustained periodic stimulation in which strong (*T_R _*= 1) pulses of 5 minute duration alternate with stimulus-free intervals of 55 minute duration. The panel at right shows the power spectral density (PSD) of the response as a function of pulsing frequency: a vertical strip cut from this panel provides a colour-coded version of the PSD of the models's response to pulsed forcing at the corresponding frequency.

Figure 4 illustrates the continuously-pulsed analogue of the experiments from Figure 1. It shows the spectral content of the model's response to a long periodic pulse train in which five minute periods of strong (*T_R _*= 1) stimulation alternate with periods during which *T_R _*= 0. The panel at left shows a typical response to this sort of strong, pulsed, periodic forcing: after a brief transient the response is periodic with the same period as the forcing pulse train. The right panel, which is a heat map showing the power spectral density of the response as a function of pulsing frequency, shows that similar entrainment occurs over a wide range of frequencies: the bright lines—which correspond to peaks in the power spectral density—have integer slopes, indicating that the power in the response is concentrated at harmonics of the forcing frequency.

**Figure 5:**
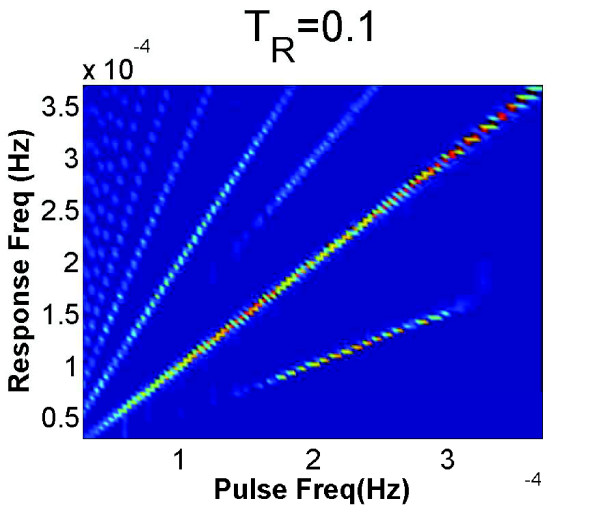
**Response to periodic trains of weak pulses**. A heat map summarising the power spectral density of NF-*κ*B localisation when the model is subjected to pulsed forcing with *T_R _*= 0.1. For forcing frequencies in the range 1.5-3.0 × 10^-4^. Hz. the lowest-lying line segment, which has slope ≈ 1/2, provides evidence of subharmonic resonance.

However, when the stimulating pulses are weaker the nonlinearity of the system leads to more complex patterns of resonance. In particular, both subharmonic resonance—when periodic forcing excites a response at a rationally-related lower frequency—and superharmonic resonance—in which a pure sinusoid excites responses containing higher harmonics—are possible. Note that these terms are defined with reference to the *forcing *frequency, a convention used in, for example, [26]. Superharmonic resonance is harder to identify when, as with the rectangular pulses used here, the periodic forcing already has power at higher harmonics, but subharmonic resonance occurs when 0.01 <*T_R _*< 0.2, as is evident in the power spectral densities summarized in Figure 5. In addition to the bright lines with integer slopes, lines with slopes $\frac{1}{2}$ and $\frac{3}{2}$ also appear. The first of these provides evidence that subharmonic resonance occurs over a wide range of pulsing frequencies.

When *T_R _*≥ 0.2 the power spectral density of the response shows power only at integer multiples of the pulsing frequency, indicating that the response is fully entrained by the forcing. Given that the pulse-strength used in the experiments corresponds to *T_R _*= 1, these modelling results are in qualitative agreement with the experimental data in Figure 1: the stimulation was so strong that we should have expected it to have entrained the responses completely.

#### Sinusoidally modulated stimulation

In this section we study the response of Horton's model to sinusoidally modulated stimulation of the form

(2) $$T_{R}(t)=\varepsilon (1+\eta \sin(2\pi \nu t)).$$

Here 0 ≤ ε ≤ 1/(1 + *η*) is the time average of the stimulus strength while 0 ≤ *η *≤ 1 and *ν *> 0 are, respectively, the relative amplitude and the frequency of the sinusoidal modulation. With this parameterization *T_R _*(*t*) has period τ = 1/*ν *and range

$$0\leq \varepsilon (1-\eta )\leq T_{R}(t)\leq \varepsilon (1+\eta )\leq 1.$$

**Figure 6:**
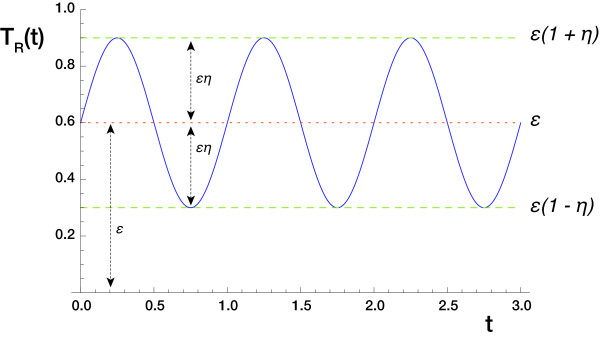
**Sinusoidal forcing function**. The function *T_R _*(*t*) for the parameter values *ν *= 1, *η *= 0.5 and *ε *= 0.6.

The roles of the parameters are illustrated in Figure 6.

The case with *η *= 1 is the straightforward substitution of pulse trains with sinusoidal waves having peak-to-trough amplitude 2ε. Our motivations for this formulation are twofold: firstly, provided *η *< 1 we have *T_R_*(*t*) > 0 at all times and so expect the system more readily to exhibit oscillations similar to those induced by constant stimulation. Secondly, periodic forcing of the form (2) converts our model into a sinusoidally forced nonlinear oscillator, a class of systems that has been studied very extensively (see, for example, Pikovsky *et al*. [27], Wiggins [26] or Nayfeh and Mook [28]).

When *η *= 0 the forcing (2) reduces to a constant stimulus with *T_R _*(*t*) = ε and the bifurcation analysis illustrated in Figure 3 leads us to expect stable oscillations when ε > 0.366 and a stable steady-state otherwise. But for *η *> 0 the system's response depends delicately on the relationship between the forcing frequency *ν *and the natural frequency *ν*_0_. When the forcing frequency *ν *and natural frequency *ν*_0 _are close (the requisite degree of closeness depends on *η*) the response will become *mode locked*, or synchronized with the forcing: it will then have the same frequency *ν *as the forcing and its power spectral density will be concentrated around the harmonics of *ν*. When the difference (*ν *-*ν*_0_) is larger—when it lies just outside of a critical interval around zero whose size depends on *η *—the response becomes *quasiperiodic *and its power spectrum has features at frequencies *f *of the form

(3) $$f=p\nu +q\nu_{0}$$

where *p *and *q *are integers.

If *pν *and *qν*_0 _are close, the system's nonlinearity will permit what is called *synchronization of order p/q*: there will be periodic responses in which the intrinsic oscillator goes through *p *cycles for every *q *periods of the forcing, so that the response has frequency

(4) $$f=p\nu \approx q\nu_{0}$$

This idea allows us to give precise definitions for the terms sub-and superharmonic resonance used above: the former corresponds to resonances where *p *= 1 and *q *> 1 in (4), while the latter corresponds to *q *= 1 and *p *> 1. Finally, when both *η *and the difference (*ν *-*ν*_0_) are large, the system's response can become chaotic, so that the features in its power spectral density bear no simple relationship to the frequencies *ν *and *ν*_0_.

**Figure 7:**
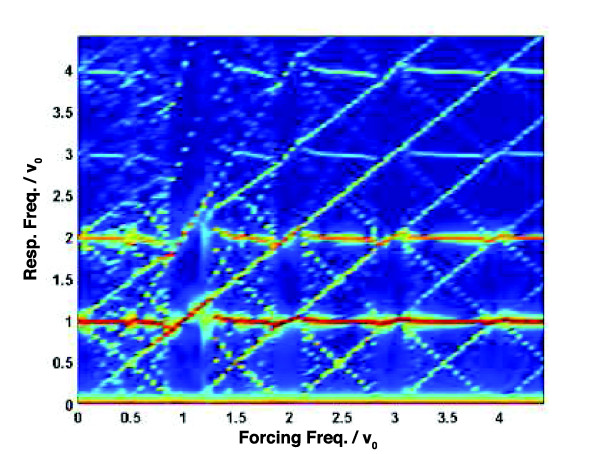
**Nonlinear resonances to sinusoidal forcing**. A heat map showing the power spectral density of the response of Horton's model to forcing of the form (2) with *ε *= *η *= 0.5 and frequencies in the range 0 ≤ *ν *≤ 4.5 × *ν*_0_.

Figure 7 which is a heat map of the power spectral density of the response generated by relatively strong sinusoidal forcing of the from (2), illustrates many of the behaviours described above. Consider first the vertical strip with *ν*/*ν*_0 _≈ 1. In this region the sinusoidal modulation entrains the NF-*κ*B system essentially completely and so the heat map resembles the corresponding region in the left panel Figure 4: the power in the response is concentrated along lines corresponding to harmonics of the forcing frequency. Away from the region *ν*/*ν*_0 _≈ 1 the power spectrum of the response is considerably more complex: the strong horizontal bands in Figure 7 which occur at integer multiples of *ν*_0_, show that there is substantial power at the NF-*κ*B system's natural frequency and its harmonics. Additionally, the nonlinearity of the system means that the response has power at frequencies given by

$$f=q\nu_{0}\pm \nu ,$$

which gives rise to the network of lines with slope of ±1. Finally, Figure 7 also exhibits sub-harmonic resonance: vertical strips for which *ν *≈ *qν*_0 _(with *q *a whole number) show power concentrated at the forcing frequency, but also at frequencies *f *= *ν/p*, where *p *is a whole number. This is perhaps clearest in the strip *ν *≈ 2*ν*_0_, where the strongest spectral feature lies along the line *f *= *ν*/2 ≈ *ν*_0_.

**Figure 8:**
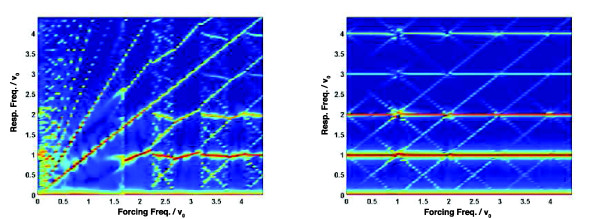
**Weak and strong modulations**. Heat maps showing the power spectral density of the responses to forcing of the form (2) with *ε *= 0.5 and *η *= 1 (left) or *η *= 0.1 (right).

The two panels of Figure 8 are analogues of Figure 7 but with especially strong (*η *= 1, left panel) or weak (*η *= 0.1, right panel) modulation. The qualitative features are much the same, though it is interesting to note that in the limit of very strong modulation—when *η *= 1 and so *T_R _*(*t*) vanishes once per forcing period—the system is very strongly entrained by the forcing and does not show much power at its natural frequency *ν*_0 _or its harmonics until the forcing frequency *ν *> 1.5 *ν*_0_. By contrast, when the modulation is weak evidence of modal interaction is also weak, with very narrow mode-locking regions near forcing frequencies of the form *ν *≈ *mν*_0_.

**Figure 9:**
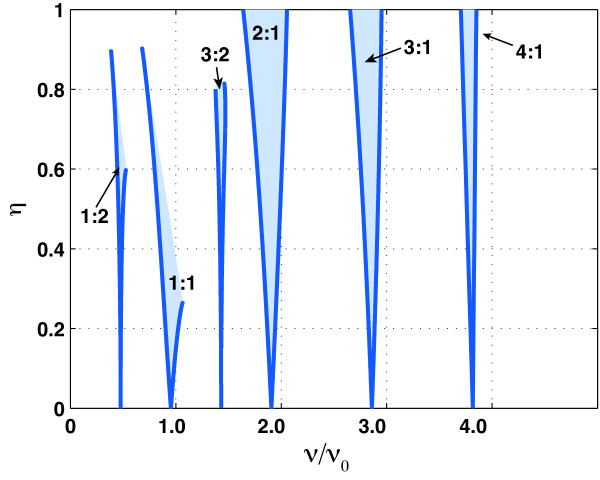
**Arnol'd tongues**. The qualitative behaviour of Horton's NF- *κ*B system when subjected to forcing of the from 2 with *ε *= 0.5: when the modulation frequency *ν *and amplitude *η *lie inside the shaded regions the response is periodic with a period that is related, by a relation like (5), to both the modulation's period and the system's natural period.

As Figures 7 and 8 illustrate, the susceptibility of our model NF-*κ*B system to resonance with sinusoidal modulations depends strongly on the amplitude of the modulation: Figure 9 provides a quantitative survey of this phenomenon. The shaded regions are examples of *Arnol'd tongues*: their precise shapes can be calculated with the methods outlined in the Appendix. When the modulation frequency and amplitude (*ν*, *η*) lie inside these tongues, the response of the system will be periodic, with a period τ that is an integer multiple of the modulation period 1/*ν *and that also lies close to an integer multiple of the natural Period 1/*ν*_0_:

(5) $$\tau =\frac{q}{\nu }\approx \frac{p}{\nu_{0}}$$

where *p *and *q *are whole numbers. Generally speaking these periodic responses—which are a nonlinear generalization of the familiar phenomenon of resonance in linear systems—are easiest to excite when the numbers *p *and *q *are small: the tongues in Figure 9 are labelled by ratios *q:p *where *p *and *q *are as in (5). Although Figure 9 illustrates this story for the specific family of modulations with *ε *= 0.5, the qualitative picture is essentially the same for all values in the range of 0.366 <*ε *< 0.5: in all cases there will be narrow tongues of parameter combinations (*ν*, *η*) for which the response is periodic with a period given by a resonance relation like (5). For modulations whose parameters lie outside the tongues the qualitative behaviour of the response will be more complex, having a power spectrum qualitatively similar to those in Figure 7, with features at harmonics of the modulation frequency, at harmonics of the natural frequency *ν*_0 _(which varies with *ε*) and at near-resonant combinations (3) of the two.

When *T*_R _< 0.366, the unforced system is a damped oscillator and so, when subjected to periodic forcing, may exhibit resonant phenomena. By using the same numerical simulation and power spectral density analyses as we did for the case *ε *= 0.5, we find that the responses of the pathway to forcing with with *T_R _*< 0.366 can be divided into two groups: those in which harmonic, sub-and superharmonic resonances can be observed (in the region 0 <*ε *≤ 0.01), and those for which only harmonic and subharmonic resonance can be observed.

We have focussed here on periodic and quasiperiodic oscillatory interactions amenable to power-spectral analyses of the sort illustrated in Figures 4, 5, 6, 7, 8 and, for single-cell recordings, in the lower panels of Figure 1. But there is every reason to expect a much richer range of dynamical behaviour: Fonslet *et al*. [20], who applied forcing of the form (2) with *η *= 1 to a simplified NF-*κ*B model, saw evidence of a period-doubling cascade as well as chaos and strange attractors. Complete analysis of these more complex dynamical regimes requires tools beyond the power spectrum, and so we defer their exploration to a future paper.

## Conclusions

Given that intrinsically nonlinear chemical kinetics underpin cell-signalling networks, one shouldn't expect these systems to be linear and any analysis of experimental time series, whether in the time or frequency domain, must take this intrinsic nonlinearity into account. Our modelling studies suggest that coupling even the simplest, sinusoidal signal into the NF-*κ*B network can give rise to a host nonlinear phenomena, including harmonic, subharmonic and superharmonic resonances as well as quasiperiodic and even chaotic behaviour.

The simulation studies reported here used an externally-imposed oscillatory forcing as a proxy for interactions between the core NF-*κ*B feedback loop and other oscillatory networks. Our results suggest that interactions between the NF-*κ*B oscillator and other oscillatory pathways can give rise to extremely rich temporal signalling programs and so, perhaps, to many distinct patterns of expression for target genes.

## Methods

### Differential Equations

The model studied in this paper is specified by the following ODEs. External forcing via TNFα stimulation is represented by the function *T_r _*(*t*), whose value ranges between 0 and 1, indicating the strength of stimulation as a fraction of the maximum possible.

$$\begin{array}{c} \text{NFk}{\text{B}}^{\prime }=k_{d1}*\text{IkBaNFkB}-k_{a1}*\text{IkBa}*Ν\text{FkB}\\ \;-k_{i1}*\text{NFkB}+k_{degc}*\text{IkBaNFkB}\\ \;+k_{e1}f*\text{nNFkB}+k_{degpin}*\text{pIkBaNFkB;}\\ \text{IkB}{\text{a}}^{\prime }=k_{d1}*\text{IkBaNFkB}-k_{a1}*\text{IkBa}*\text{NFkB}\\ \;-k_{i2}*\text{IkBa}+k_{e2}*\text{nIkBa}\\ \;-k_{degf}*\text{IkBa}+k_{tria}*\text{tIkBa}\\ \;-k_{c2}*\text{IKK}*\text{IkBa;}\\ \text{IkBaNFk}{\text{B}}^{\prime }=k_{a1}*\text{IkBa}*\text{NFkB}-k_{d1}*\text{IkBaNFkB}\\ \;+k_{elc}*\text{nIkBaNFkB}-k_{degc}*Ι\text{k}Β\text{aNFkB}\\ \;-k_{c3}*\text{IKK}*\text{IkBaNFkB;}\\ \text{nNFk}{\text{B}}^{\prime }=k_{d\text{1}}\;\text{*}\;\text{nIkBaNFkB}\;-\;k_{a1}*\text{nIkBa}*\text{nhNFkB}\\ \;+k_{v}*k_{i1}*\text{NFkB}+k_{degcn}*\text{nIkBaNFkB}\\ \;-k_{v}*k_{e1f}*\text{nNFkB};\\ \text{nIkB}{\text{a}}^{\prime }=k_{d1}*\text{nIkBaNFkB}-k_{a1}*\text{nIkBa}*\text{n}Ν\text{FkB}\\ \;+k_{v}*k_{i2}*\text{IkBa}-k_{v}*k_{e2}*\text{nIkBa}\\ \;-k_{def}f*\text{nIkBa;}\\ \;\text{nIkBaNFk}{\text{B}}^{\prime }=k_{a1}*\text{nIkBa}*\text{nNFkB}-k_{d1}*\text{nIkBaNFkB}\\ \;-k_{v}*k_{elc}*\text{nIkBaNFkB}\\ \;-k_{degcn}*\text{nIkBaNFkB};\\ \;\;\text{tIkB}{\text{a}}^{\prime }=k_{ctria}-k_{deg\text{​}tia}*\text{tIkBa}\\ \;\;+k_{irtia}*\left(\frac{{\text{nNFkB}}^{h}}{{\text{nNFkB}}^{h}+k^{h}}\right)\;;\\ \text{IKK}{\text{n}}^{\prime }=k_{p}*\text{IKKi}*\left(\frac{k_{bA20}}{k_{bA20}+\text{A20}*T_{r}}\right)\\ \;-T_{r}*k_{a}*\text{IKKn};\\ \text{IK}{\text{K}}^{\prime }=T_{r}*k_{a}*\text{IKKn}-k_{i}*\text{IKK;}\\ \text{IKK}{\text{i}}^{\prime }=k_{i}*\text{IKK}\\ \;-k_{p}*\text{IKKi}*\left(\frac{k_{bA20}}{k_{bA20}+\text{A20}*T_{r}}\right);\\ \text{tA2}0^{\prime }=k_{ctra}-k_{degta}*\text{tA20}\\ \;+k_{itra}\text{*}\left(\frac{{\text{nNFkB}}^{h}}{{\text{nNFkB}}^{h}+k^{h}}\right);\\ \text{A2}0^{\prime }=k_{tra}*\text{tA20}-k_{da}*\text{A20};\\ \text{pIkB}{\text{a}}^{\prime }=k_{c2}*\text{IKK}*\text{IkBa}-k_{degpi}*\text{pIkBa;}\\ \text{pIkBaNfk}{\text{B}}^{\prime }=k_{c3}*\text{IKK}*\text{IkBaNFkB}\\ \;-k_{degpin}*\text{pIkBaNFkB;} \end{array}$$

### Initial conditions

All the numerical experiments reported above began with the initial concentrations

IkBaNFkB=0.1042424 and IKKn=0.1042424

while all others start at zero. We then integrated the ODEs through 2000 minutes of simulated time and used the result as an initial condition for the various TNFα-stimulation studies.

### Numerics

The bifurcation diagram in Figure 3 and the plot of the Arnol'd tongues in Figure 9 were prepared using Bard Ermentrout's XPPAUT [29], a front end for the powerful numerical bifurcation package AUTO [30]. All our other numerical work used MATLAB^© ^to integrate the ODEs, compute the power spectra and plot the figures.

## Authors' contributions

YW did the numerical experiments reported here, basing her work on code originally written by CAH, who also developed the underlying model. PP and CAH provided biologically-informed advice on modelling, especially on how best to couple the model to an external signal. MRHW, DBK and DSB provided critical readings of the manuscript, as well scientific guidance throughout the project. MRM worked closely with YW on the computational side and, with YW, wrote the first draft and prepared the figures. All authors reviewed and approved the final draft.

## Appendix: circle maps

Here we discuss a standard mathematical tool, the *circle map*, used to study the response of a nonlinear oscillator subjected to periodic forcing. The idea is to consider sufficiently weak forcing that the response is close to that of the unforced system, and then define a phase angle *ϕ *that is close to the phase of the corresponding unforced oscillator.

If *ω*_0 _is the frequency of the unperturbed oscillator and *ω *is the forcing frequency then, after one period of the forcing, *ϕ *will have advanced by

$$\begin{array}{c} \Delta \phi =\omega_{0}\tau \;+\;\varepsilon G(\phi )\\ =\omega_{0}\left(2\pi /\omega \right)+\varepsilon G(\phi ) \end{array}$$

where *τ *= 2*π/ω *is the period of the forcing, *ε *is a measure of its amplitude and G(*ϕ*) is a phase-dependent function that characterizes the response.

The simplest circle map, studied here by way of illustration, is given by a function *F *: [0, 2*π*) → [0, 2*π*) of the form of

(6) $$\begin{array}{c} F(\phi )=\phi +\Delta \phi \\ =\phi +\omega_{0}\tau +\varepsilon \sin(\phi )\\ =\phi +2\pi \alpha +\varepsilon \sin(\phi ), \end{array}$$

where *α *= (*ω*_0_/*ω*), and 0 <*ε *≪ 2*π*. The dynamics of a circle map can be characterized with a single parameter called the *rotation number*. For a given initial point *ϕ*_0_, the rotation number is defined as the long-term average phase shift per one iteration (that is, per one period of the forcing):

(7) $$\rho (\phi_{0})=\underset{n\to \infty }{\lim}\;\frac{\phi_{n}-\phi_{0}}{2\pi n},$$

where

$$\phi_{n}=F^{n}(\phi_{0})=F\left(F^{n-1}(\phi_{0})\right)=F\left(F\left(F^{n-2}\left(\phi_{0}\right)\right)\right)....$$

One can show that the limit in the definition of the rotation number (7) exists and does not depend on the initial point *ϕ*_0 _[31], p.102. The qualitative dynamics of the forced system are thus of two types: motions with rational rotation numbers and those with irrational ones. Further, the rotation number is retional-say $\rho =\frac{p}{q}$, with *p *∈ ℕ and *q *∈ ℤ^+ ^—if and only if there exists some *ϕ*_0 _∈ [0, 2*π*) such that

(8) $$\phi_{0}+2\pi p=F^{q}\left(\phi_{0}\right).$$

That is, rational rotation numbers correspond to periodic dynamics: the orbit of *ϕ*_0 _is called a *(p, q) cycle*. On the other hand, irrational rotation numbers correspond to quasiperiodic dynamics [27,31,32]. 

According to a theorem of Denjoy [33], if the rotation number is irrational there is a continuous, invertible change of coordinates *h*: [0, 2*π*) → [0, 2*π*), say, *h*(*ϕ*) = *θ*, such that

$$h\left(F\left(\phi \right)\right)=h\left(\phi \right)+2\pi \rho \;\text{or}\;\tilde{F}\left(\theta \right)=\theta +2\pi \rho$$

Where $\tilde{F}\left(\theta \right)\equiv h\left(F\left(h^{-1}\left(\theta \right)\right)\right)$. This implies that forced oscillators whose corresponding circle maps have irrational rotation number never repeat periodically.

Results of Arnold [31] show that the qualitative behaviour of the circle map (6) is stable against arbitrary small perturbations if and only if the rotation number is rational. We can thus expect that if *F *has a rational rotation number $\frac{p}{q}$, there exists a region of parameter values (*α*, *ε*) such that the all the forced systems whose parameters lie in this region share the same rational rotation number: that is, they all have the same sorts of periodic response. Such regions of parameter values are called *Arnol'd tongues*.

 A similar story holds for the sinusoidally forced NF-*κ*B system: when the time-average of the stimulation is sufficiently strong that the system supports periodic oscillations with frequency *ω*_0_, and when the amplitude of the forcing is sufficiently weak that it is sensible to measure a phase *ϕ *with respect to that of the intrinsic oscillator, then one can construct numerically a circle map of the form

(9) $$F^{\prime }\left(\phi \right)=\phi +\omega_{0}T+\eta G^{\prime }\left(\phi \right),$$

where *T *= 2*π*/*ω *is the period of the forcing. Although the function *G' *in (9) is not as simple as the the *G *(*ϕ*) in (6), the map *F' *still has (*p*, *q*) periodic responses and a corresponding system of Arnol'd tongues: Figure 9 shows examples.

We'll conclude this Appendix by explaining why, at least for the simple circle map (6), the Arnol'd tongues are wedge-shaped. Consider the case where *F *has rational rotation number $\rho =\frac{p}{q}$. Then, as mentioned above, there exists a periodic point *ϕ*_0 _satisfying (8). If we expand *F^q ^*(*ϕ*_0_) in powers of *ε *we find

$$\begin{array}{c} F^{q}\left(\phi_{0}\right)=\phi_{0}+q2\pi \alpha \\ +\varepsilon \left[\sin\left(\phi_{0}\right)+\sin\left(\phi_{1}\right)+\cdots +\sin\left(\phi_{q}{}_{-\;1}\right)\right]\\ +\varepsilon^{2}\left[\cdots \right]+\cdots \end{array}$$

Consider the case where *α *is very close to a rational number: $\alpha =\frac{p}{q}+\beta$ with |*β*| ≪ 1/*q*. Then

$$F^{q}\left(\phi_{0}\right)\approx \phi_{0}+2\pi p+G_{p/q}\left(\beta ,\varepsilon ,\phi_{0}\right),$$

Where

(10) $$\begin{array}{l} G_{p/q}\left(\beta ,\varepsilon ,\phi_{0}\right)=\\ \;\;\;2\pi q\beta +\varepsilon \;\left[\sin\left(\phi_{0}\right)+\cdots +\sin\left(\phi_{q}{}_{-1}\right)\right]\\ \;\;\;2\pi q\beta +\varepsilon \;\left[\sin\left(\phi_{0}\right)+\cdots +\sin\left(F^{q-1}\left(\phi_{0}\right)\right)\right] \end{array}$$

It is not hard to show that *G_P/q _*is bounded and continuous when regarded as a function of *ϕ*_0 _and so attains its maximum and minimum values on [0, 2*π*]. Thus, for each fixed *ε*, there is an interval of *β *on which *G_P/q _*= 0 for some *ϕ *∈ [0, 2*π*]. Now consider the way in which the end points of this interval depend on *ε*. The case *q *= 1 and *p *= 0 is especially clear: Eqn. (10) becomes

$$G_{p/q}\left(\beta ,\varepsilon ,\phi \right)=2\pi \beta +\varepsilon \sin\left(\phi \right)=0$$

which has some solutions *ϕ *∈ [0, 2*π*] provided that |2*πβ*| ≤ *ε *or |*β*| ≤ *ε*/2π.

The values *q *= 1 and *p *= 1 correspond to the case where the periodic response has the same frequency as the forced system and thus undergoes one complete phase-rotation per period of the forcing. The analysis sketched above shows that this particular form of mode-locking will pertain for (*ε*, *α*) in a wedge-shaped region whose width increases linearly with forcing amplitude *ε*. For more general mode-locking resonances, where *q *≥ 2, Arnol'd showed in [31] that the width of the tongue satisfies:

$$\Delta \alpha \propto \varepsilon^{q}.$$
